# Re-interpretation of the mechanism of type 2 diabetes mellitus based on a framework of psychosomatic medicine: a real-world study

**DOI:** 10.1186/s12888-022-04315-1

**Published:** 2022-11-08

**Authors:** Wenjiao Min, Bo Zhou, Zhengyu Li, Nie Tang, Xu Zhang, Jinxiang Wang, Yuexin Chen, Yaling Zhou, Ruhan A, Lei Tang, Gang Li, Xueli Sun

**Affiliations:** 1 Psychosomatic department, Sichuan Provincial People’s Hospital, University of Electronic Science and Technology of China, Sichuan Provincial Center for Mental Health, Chinese Academy of Sciences Sichuan Translational Medicine Research Hospital, 610072 Chengdu, People’s Republic of China; 2 Mental Health Center, West China University Hospital, Sichuan University, 610041 Chengdu, People’s Republic of China; 3 Department of Gynecology and Obstetrics, West China Second University Hospital, Sichuan University, Chengdu, People’s Republic of China; 4 Department of endocrinology, Sichuan Provincial People’s Hospital, Chengdu, People’s Republic of China; 5 Department of Psychology, Xinxiang Medical College, Henan Province Xinxiang, People’s Republic of China; 6 Mental Health Center, North Sichuan Medical College, Sichuan Province Nanchong, People’s Republic of China; 7 The Third People’s Hospital of Tianshui, Gansu Province Tianshui, People’s Republic of China

**Keywords:** Bipolar disorder, Dysrhythmia, Glucose metabolism, Rhythm, Type 2 diabetes mellitus

## Abstract

**Objective:**

Using bipolar disorder (BD) as a control, we explored the possible developmental process of impaired glucose metabolism rhythm.

**Methods:**

In total, 441 subjects (77, 162, 134, 54, and 14 in the pre-diabetes [pre-DM], DM, BD, BD + pre-DM, and BD + DM groups, respectively) and 160 controls were included. All subjects were assessed using the Neuroticism Extraversion Openness Five-Factor Inventory (NEO-FFI). The hypothalamic-pituitary-adrenal (HPA) and hypothalamic–pituitary–thyroid (HPT) axes were measured.

**Results:**

Cluster analysis showed that the BD, BD + DM, and DM groups were classified as the ‘disease group, the BD + pre-DM group as the ‘mixed period group’, and the pre-DM group as the ‘pre-disease group’. The conscientiousness factor scores of the NEO-FFI in the ‘disease group’ were higher than the norm but lower than the norm in the ‘pre-disease group’. The scores of neurotic factors in the ‘pre-disease’ and ‘mixed period’ groups were both significantly higher than that in the ‘disease group’ (corrected *p* < 0.001). The incidences of the abnormal HPA axis decreased gradually from the ‘pre-disease group’ to the ‘mixed period group’ then to the ‘disease group’, while those of the HPT axis slightly increased at first and then significantly decreased. The overall prediction rate of the multiple logistic regression model was 92.7%.

**Conclusion:**

This study suggests that progression of pre-diabetes to DM is a continuous process from local abnormalities to rhythm disorder of glucose metabolism. This understanding can be applied to the whole course management and early intervention of DM and to the future development of optimised treatment based on rhythm regulation.

**Trial registration:**

Clinical trial registration number: ChiCTR1800019064. Name of trial registration: Identify and the optimization of treatment for non-infectious chronic diseases under the “stress-dysrhythmia” theory hypothesis (Registration date: 24/10/2018). The full trial protocol can be accessed at the Chinese Clinical Trial Registry (http://www.chictr.org.cn/).

**Supplementary Information:**

The online version contains supplementary material available at 10.1186/s12888-022-04315-1.

## Introduction

Based on the biopsychosocial model, psychosomatic medicine, which has paid great attention to the integrity of the body and disease, believes that the development of chronic non-infectious diseases, including type 2 diabetes mellitus (DM), is closely related to psychosocial factors [1]. However, it is difficult to systematically describe the progression of these diseases, and current diagnostic criteria and treatment guidelines do not fully explain the comorbidities and pathophysiological changes. For example, patients with DM often have insomnia, depression, or anxiety [2]. In addition, current drugs for DM treatment mainly focus on the control of blood glucose levels and have no direct effect on the prevention of symptoms of comorbidities and complications [3].

The previous study preliminarily puts forward the ‘stress-dysrhythmia’ theoretical hypothesis by recognising atypical symptoms and optimising the treatment of bipolar disorder (BD) [4]. According to this hypothesis, under the action of various internal and external stress factors, individuals would probably exhibit local abnormal physical or mental rhythms based on different genetic backgrounds, such as situational insomnia and gestational DM. This stage could be called the ‘prophase of rhythm disorder’. If the intervention is not timely or the stress persists, a certain rhythm of the body may be completely disturbed, entering the ‘rhythm disorder’ stage, such as DM, BD, and hypertension. Dysrhythmia may be difficult to reverse if it develops further and enters the ‘loss of rhythm’ stage, such as malignant tumours and serious complications of DM. This hypothesis is based on the theory and practice of psychosomatic medicine and breaks the traditional definition of diseases, attaching importance to the role of biological rhythms in body health.

In recent years, increasing attention has been paid to the role of rhythm disturbances in the development of diseases. For example, patients with BD or DM may have disturbance of hormone secretion rhythm of the neuroendocrine axes, including the hypothalamic-pituitary-adrenal (HPA) axis, and stress response caused by which has been considered a contributing factor in the comorbidity of both [5]. The risk of DM in CLOCK gene rs4580704 CC genotype carriers is significantly higher than that in carriers of other genotypes [6], and patients with BD carrying the rs1801260 C allele have severe insomnia, higher recurrence rate, and poorer treatment response than those with the other genotypes [7].

According to the ‘stress-dysrhythmia’ theoretical hypothesis, DM, which represents the glucose metabolism disorder, and BD, which represents the emotional rhythm disorder, should both belong to the ‘rhythm disorder’ as they share a similar pathophysiological basis. In addition, the process of abnormal glucose tolerance to type 2 DM and its complications should be a continuous developmental process from the ‘prophase of rhythm disorder’ to the ‘rhythm disorder’ and then to the ‘loss of rhythm’ stage. However, sufficient evidence to support this hypothesis is lacking.

Based on real-world research data, this study compared BD with prediabetes and DM to explore the abnormal developmental process of glucose metabolic rhythm and to identify markers for the definition of rhythm staging. This study aimed to preliminarily verify the ‘stress-dysrhythmia’ theoretical hypothesis under a framework of psychosomatic medicine, to propose a new theory of rhythm related to the pathogenesis of DM, to improve whole-process management, and to optimise treatment with a holistic health view.

## Methods

### Subjects

A total of 441 subjects with BD, type 2 DM, or pre-diabetes (including impaired fasting blood glucose [IFG] or impaired glucose tolerance [IGT]) were enrolled from southwest China between December 2018 and December 2019. Subjects with BD fulfilled the Diagnostic and Statistical Manual of Mental Disorder, Fourth Edition (DSM-IV) criteria, being free of any medication, or having not taken psychotropic medication within three months before enrollment. The diagnosis was independently assigned by two senior psychiatrists administering the Structured Clinical Interview for DSM-IV Disorders-Clinician Version (the Kappa value of the consistency check was > 0.8). Patients with DM fulfilled the World Health Organization (WHO)’s 1999 diagnostic criteria for type 2 DM. Patients with pre-diabetes (pre-DM) fulfilled the WHO’s 1999 diagnostic criteria for IFG or IGT. The exclusion criteria were as follows: BD group: (1) other Axis I psychiatric disorders or Axis II disorders; (2) any history of major medical/neurological disorders; (3) use of any drugs that may affect metabolism, including glucose levels, in the past 3 months; and (4) pregnancy or lactation; DM and pre-diabetes groups: (1) type 1 DM or other special types of DM; (2) acute complications or severe chronic complications; (3) use of β blockers or glucocorticoids in the past 3 months; (4) history of other endocrine or autoimmune diseases; (5) history of major medical/neurological disorders; (6) history of organic mental disorders, substance abuse, and schizophrenia, including its spectrum disorders; and (7) pregnancy or lactation.

We enrolled 160 race-matched healthy people as controls (mean age of 42.86 ± 11.88 years, 63 males and 97 females). We ruled out current or past serious physical illnesses or DSM-IV Axis I disorders through the Structural Clinical Interview for DSM-IV Axis I Disorders, non-patient edition [8].

All subjects were of unrelated (no blood relationship) Chinese Han origin, sharing similar geographic and sociodemographic data. This study was approved by the China Ethics Committee of Registering Clinical Trials in the West China Hospital of Sichuan University (Ethic Review No. ChiECRCT-20,180,187). Written informed consent was obtained from all participants.

### Data collection

Demographic and clinical data were collected from each subject after enrolment. The Neuroticism Extraversion Openness Five-Factor Inventory (NEO-FFI) was used for personality assessment.

### Hormone measurements

Ten millilitres of peripheral venous blood were collected from each subject for hormone detections of the HPA and hypothalamic–pituitary–thyroid (HPT) axes at 8:00 am on the next day after enrolment. The seven detected hormones were as follows: cortisol (COR), adrenocorticotrophic hormone (ACTH), thyrotropin-stimulating hormone (TSH), 3-triiodothyronine (TT3), thyroxine (TT4), free triiodothyronine (FT3), and free thyroxine (FT4). COR, T3, T4, FT3, and FT4 levels were measured by electrochemiluminescence quantitative assays. TSH was measured using the electrochemiluminescence double-antibody sandwich method. ACTH was measured using radioimmunoassay. An individual with at least one abnormal value of ACTH and COR was considered to have an abnormal HPA axis, while an individual with at least one abnormal value of TSH, TT3, FT3, TT4, and FT4 was considered to have an abnormal HPT axis.

### Statistical analyses

Sociodemographic data were analysed using Pearson’s χ^2^ test or one-way analysis of variance (ANOVA). In addition, the comparison of the factor scores of the NEO-FFI or hormone levels among the different groups was performed using ANOVA with the Bonferroni correction for multiple comparisons. Comparisons of the abnormal rates of the HPA or HPT axis among the groups were performed using the χ^2^ test. Logistic regression was used to construct a multi-factor model, while hierarchical cluster analysis was used to further cluster the groups. All tests were two-tailed, with alpha set at 0.05. Analysis was performed using SPSS18.0.

## Results

The subjects were divided into five groups based on comorbidities: 77 patients in the pre-DM group, 162 in the DM group, 134 in the BD group, 54 in the BD + pre-DM group, and 14 in the BD + DM group. We referred to the DM and pre-DM groups as the ‘abnormal glucose metabolism group’ (239 patients) and the BD + pre-DM and BD + DM groups as the ‘comorbidities group’ (68 patients). The comorbidity rate of abnormal glucose metabolism in patients with BD was 33.7% in this study.

### Comparisons of clinical data among the five groups

#### Clinical data

There were significant differences in sex, age of onset of DM, pre-DM and BD, marital status, educational level, family history, hypertension comorbidity rate, and stable income among the five groups (corrected *p* < 0.01, Supplementary Table 1).

#### NEO-FFI scores

The scores of the five factors of the NEO-FFI were also significantly different among the five groups (*p* < 0.001, Supplementary Table 2). The conscientiousness factor score in the DM group was significantly higher than the norm, while it was significantly lower in the pre-DM group.

The agreeableness factor scores decreased from the BD group (46.28 ± 4.141) to the abnormal glucose metabolism group (44.78 ± 7.853) and then to the comorbidities group (41.30 ± 4.945) (*p* < 0.001). The conscientiousness factor score in the comorbidities group (38.81 ± 6.873) was significantly lower than that in the BD (42.64 ± 5.885) and abnormal glucose metabolism (43.80 ± 10.363) groups (corrected *p* = 0.01 for the former, 0.00 for the latter).

#### Neuroendocrine axes

There were significant differences in the levels of the seven detected hormones among the five groups (TSH: *p* = 0.019, TT3: *p* = 0.002, and *p* < 0.001 for the others; Supplementary Table 3). Besides TSH (*p* = 0.521), the levels of the other six hormones were significantly different between the control and case groups (*p* < 0.001), where the levels of FT3 and FT4 were decreased, while those of ACTH and COR increased in the case groups. The incidences of the abnormal HPT or HPA axis and biax among the five groups were also significantly different (*p* < 0.001) and were all higher than those in the control group (Supplementary Fig. 1). The incidences of the abnormal HPT and HPA axis in the BD and DM groups were both significantly lower than those in the pre-DM group (corrected *p* < 0.001).

### Logistic regression and cluster analysis for the five groups

We constructed a multiple logistic regression where the five groups were taken as dependent variables, the classification variables with significant differences among the five groups were taken as independent variables, and the numerical variables were taken as covariates. The *p*-values of the likelihood ratio test for 11 indicators, including the five factor scores of the NEO-FFI and levels of TT3, TT4, FT3, FT4, ACTH, and COR, were < 0.01, with no collinearity by multi-collinearity test (tolerance value > 0.1, Variance inflation factor (VIF) < 10).

**Fig. 1:**
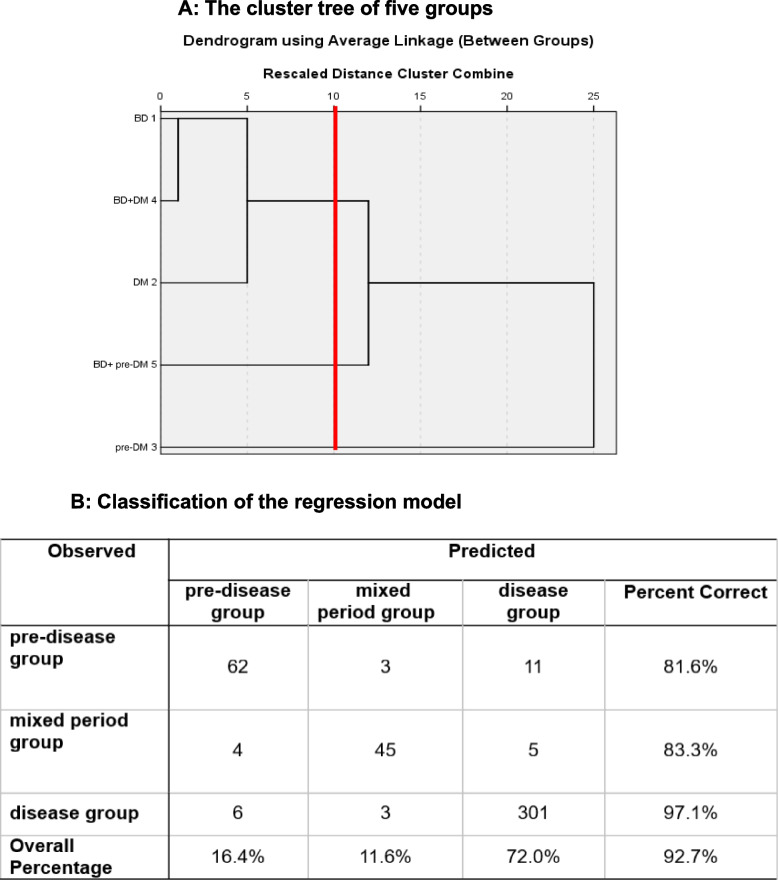
The cluster tree and regression model. **A **The cluster tree of five groups. **B** Classification of the regression model

Cluster analysis including the above 11 indicators showed that the BD, BD + DM, and DM groups were classified as one group (named as the ‘disease group’), the BD + pre-DM group classified as one group (named as the ‘mixed period group’), and the pre-DM group as one group (named as the ‘pre-disease group’) (Fig. 1A).

### Comparisons of the three groups after clustering

#### Clinical data

The comorbidity of hypertension among the three groups was significantly different (*p* < 0.001), being significantly higher in the disease group than in the pre-disease and mixed period groups (corrected *p* = 0.04 and 0.000, respectively). In addition, patients in the disease group showed a significantly higher rate of positive DM and BD family history (*p* < 0.001), while the married status was found to be significantly higher in the pre-disease group than in the mixed and disease groups (corrected *p* = 0.000 and 0.01, respectively) (Table 1).

**Table 1:** Comparisons of demographic data among the three groups after clustering

**Groups**		**Pre-disease group** **(** ***n*** ** = 77)**	**Mixed period group** **(** ***n*** ** = 54)**	**Disease group** **(** ***n*** ** = 310)**	**F/χ**^**2**^	**P**
**Variables**						
Onset age (years)		37.95 ± 11.391	27.57 ± 10.930	39.95 ± 18.598	23.877	0.000
Age at enrolment (years)		42.86 ± 11.482	34.56 ± 13.130	46.31 ± 18.927	21.759	0.000
Confirmed time (months)		32.72	16.61	33.88	5.960	0.051
sex	male	37(48.1%)	22(40.7%)	121(39.0%)	2.077	0.354
	female	40(51.9%)	32(59.3%)	189(61.0%)		
Address	town	61(79.2%)	37(68.5%)	240(77.4%)	2.381	0.304
	village	16(20.8%)	17(31.5%)	70(22.6%)		
Marriage	single	14(18.2%)	27(50%)	120(38.7%)	16.046	0.000
	married	63(81.8%)	27(50%)	190(61.3%)		
Educational level	≤ high school	51(66.2%)	27(50%)	204(65.8%)	5.196	0.074
	≥ junior college	26(33.8%)	27(50%)	106(34.2%)		
Stable income	no	37(48.1%)	28(51.9%)	112(36.1%)	7.165	0.028
	yes	40(51.9%)	26(48.1%)	198(63.9%)		
Comorbid hypertension		10(13.0%)	1(1.9%)	90(29.0%)	129.771	0.000
Family history		5(6.5%)	10(18.5%)	84(27.1%)	20.274	0.000

#### NEO-FFI scores

The five factor scores of the NEO-FFI were significantly different among the three groups (*p* < 0.01, Table 2), with the conscientiousness factor scores in the disease group being higher than the norm, whereas those in the pre-disease group being lower than the norm. In addition, the scores of neurotic factors in the pre-disease and mixed period groups were both significantly higher than those in the disease group (corrected *p* < 0.001).

**Table 2:** Comparisons of the scores of five factors of NEO-FFI among the three groups after clustering

**Factors**	**Neuroticism**	**Extroversion**	**Openness**	**Agreeableness**	**Conscientiousness**
**Groups**					
pre-disease group	36.9 ± 7.434	34.46 ± 6.754	32.69 ± 5.581	36.31 ± 7.884	33.26 ± 10.026 ↓
mixed period group	37.74 ± 9.580	38.18 ± 9.147	37.11 ± 5.958	40.17 ± 4.575	37.28 ± 6.251
disease group	29.37 ± 8.394	37.61 ± 7.041	35.69 ± 3.672	47.57 ± 3.943	45.96 ± 6.536 ↑
F	40.704	6.405	19.506	186.883	112.172
P	0.000	0.002	0.000	0.000	0.000

#### Neuroendocrine axes

There were significant differences in the levels of the seven detected hormones among the three groups (TSH, *p* = 0.028; FT3, *p* = 0.002; ACTH, *p* = 0.042; and *p* < 0.001 for the others; Table 3). Compared with the control group, the levels of FT3 and FT4 in the three groups were all decreased, while the levels of ACTH and COR were all increased.

**Table 3:** Comparisons of hormone levels and abnormal rates among the three groups after clustering

**Groups**		**Pre-disease group**	**Mixed period group**	**Disease group**	**F/χ**^**2**^	**P**	**Controls**
**Variables**							
TSH	levels	2.48 ± 1.537	2.61 ± 3.399	2.16 ± 1.706	7.153	0.028	2.28 ± 1.28
	AR^a^	9.10%	14.80%	6.80%	4.073	0.130	8.12%
TT3	levels	1.64 ± 0.575	1.5 ± 0.477	1.42 ± 0.421	15.537	0.000	1.63 ± 0.25
	AR^a^	7.80%	37.00%	8.10%	39.044	0.000	5.63%
FT3	levels	4.70 ± 1.214	4.49 ± 1.108	4.2 ± 1.136	6.485	0.002	4.97 ± 0.69
	AR^a^	19.50%	16.70%	8.10%	10.062	0.007	3.13%
TT4	levels	105.51 ± 32.832	67.59 ± 37.517	89.6 ± 23.189	36.596	0.000	96.03 ± 16.26
	AR^a^	6.50%	27.80%	5.50%	30.175	0.000	0.63%
FT4	levels	14.70 ± 4.129	13.36 ± 9.572	12.74 ± 2.969	20.132	0.000	16.90 ± 2.94
	AR^a^	18.20%	31.50%	3.90%	47.377	0.000	3.75%
AR of HPT axis		39%	57.40%	21.60%	33.074	0.000	15.63%
ACTH	levels	36.79 ± 33.077	42.52 ± 33.421	30.42 ± 22.204	6.362	0.042	24.66 ± 11.89
	AR^a^	18.20%	7.40%	10.30%	4.736	0.094	0.63%
COR	levels	656.74 ± 311.641	577.35 ± 234.275	407.56 ± 170.571	75.555	0.000	386.77 ± 109.85
	AR^a^	39.00%	35.20%	8.40%	55.256	0.000	1.88%
AR of HPA axis		45.50%	35.20%	18.10%	28.164	0.000	2.5%
AR of biax		14.30%	22.20%	3.50%	28.208	0.000	0.6%

^a^abnormal rates

Table 3 shows that the incidences of the abnormal HPT and HPA axis and biax among the three groups were also significantly different (*p* < 0.001). In addition, all incidences of the abnormal neuroendocrine axes in the case groups were higher than those in the control group.

**Fig. 2:**
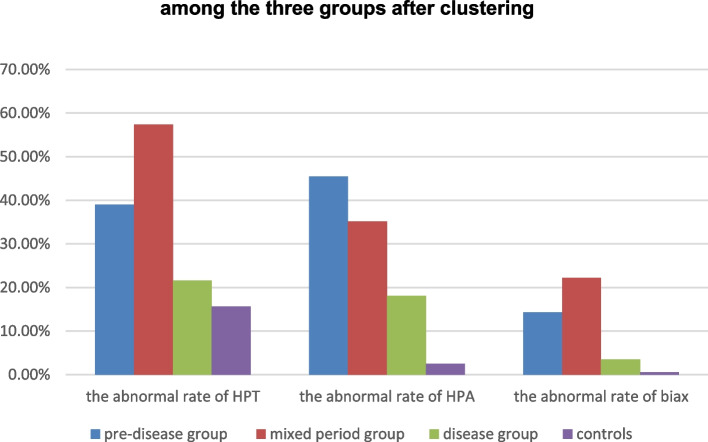
Comparisons of abnormal rates of neuroendocrine axes among the three groups after clustering

From the pre-disease group to the mixed period group and then to the disease group, the incidence of the abnormal HPA axis decreased gradually, while the incidences of the abnormal HPT axis and biax increased slightly at first and then significantly decreased (Fig. 2).

### Logistic regression for the three groups after clustering

In multiple logistic regression, where the three groups were taken as dependent variables, the classification variables with significant differences among the three groups were taken as independent variables and the numerical variables as covariates, the fitting significance level of the regression model was 0.000 and the maximum pseudo-*R*-square value of the model was 0.833, suggesting a good fitting degree. Eleven indicators, including the neuroticism, agreeableness, and conscientiousness factor scores of the NEO-FFI; levels of TT3, TT4, ACTH, and COR; and abnormity of TT4, FT4, COR, and HPA axis, were finally entered into the regression, with *p*-values of likelihood ratio test < 0.05 and without multicollinearity.

Taking the ‘disease group’ as control, the regression model of the ‘pre-disease group’ and ‘mixed period group’ was, respectively, as follows:

- G1 = LOG (P(pre-disease) / P(disease)] = 6.039 + 0.08*neuroticism score – 0.344*agreeableness score – 0.078 × conscientiousness score + 0.444*TT3 level – 0.007*TT4 level + 0.03*ACTH level + 0.006* COR level + 2.985*TT4 abnormality (0: normal; 1: abnormal) – 3.224*FT4 abnormality + 1.136*COR abnormality + 0.346*HPA axis abnormality.
- G2 = LOG[P(mixed period) / P(disease)] = -0.191 + 0.084 × neuroticism score – 0.253*agreeableness score – 0.173*conscientiousness score + 2.951*TT3 level – 0.08*TT4 level + 0.07*ACTH level + 0.004* COR level + 3.892*TT4 abnormity – 4.405*FT4 abnormity – 20.312*COR abnormity + 22.368*HPA axis abnormality.

The predictions for each stage of the above models are shown in Fig. 1B. The prediction rates for the pre-disease, mixed, and disease stages were 81.6%, 83.3%, and 97.1%, respectively. The overall prediction rate of the model was 92.7%.

## Discussion

Biological rhythms are basic characteristics of life activities. It is believed that the suprachiasmatic nucleus is the rhythm centre of the body, which maintains the coordination of the internal and external environment through the precise regulation of the ‘transcription-translation-feedback’ loop. The body may have various abnormal rhythmic activities under dysregulation, leading to the occurrence of diseases [9].

To the best of our knowledge, this study provides preliminary exploration of the rhythmic developmental stages of abnormal glucose metabolism, breaking the traditional definition of diseases. The results were consistent with the ‘stress-dysrhythmia’ theoretical hypothesis and verified that the transition from IFG or IGT to DM was a result of local abnormality of glucose metabolic rhythm to dysrhythmia. To some extent, it could explain the comorbidities [2] and dynamic conversions of different diseases [10] that are observed in clinical practice.

It was shown that patients’ social functions, such as marital status, were gradually impaired with the aggravation of abnormal glucose metabolism. Compared with patients with pre-diabetes (pre-dysrhythmia), those with DM who were in the stage of dysrhythmia had a higher positive family history of DM, which may be a risk factor for DM. Meanwhile, the molecular circadian clock is crucial in blood pressure (BP) control and patients with hypertension have a disrupted circadian BP rhythm [11]. The comorbidities of hypertension in the DM stage increased significantly, suggesting that the body may have more rhythmic abnormalities with the development of impaired glucose metabolism rhythm.

Neuroticism is a prominent personality trait in patients with pre-diabetes. Neurotic individuals tend to have poor emotional regulation ability and often show stronger responses to various stresses, leading to insomnia, anxiety, or depression [12]. Therefore, neurotic personality might be a susceptibility trait for early somatic warning of stress. In contrast, patients with type 2 DM had prominent conscientious personality traits, which was consistent with the findings of our previous study and another study [13, 14]. Conscientious individuals often show diligence, self-discipline, and a sense of responsibility. However, high conscientiousness may lead to a reverse damaging effect on life satisfaction under certain stressful conditions [15], suggesting that these individuals have poor mental resilience in response to stress and might have alexithymia, leading to abnormal body rhythms [16]. Changes in individual personality traits from pre-diabetes to type 2 DM may be a risk factor for predicting the developmental stage of abnormal glucose metabolism rhythm.

Professor Chrousos has suggested that all organisms maintain a homeostasis, which is constantly challenged by internal or external stressors. Stress occurs When homeostasis is threatened. The body’s stress system mediates the stress response, among which, neuroendocrine hormones including corticotropin-releasing hormone in the HPA axis play an important role in the regulation of basal homeostasis and response to threats, and are involved in the pathogenesis of metabolic diseases [17]. The HPA axis is believe to be closely related to body growth and development, and its regulatory changes play a key role in life extension [18]. A lower HPA axis activity has been shown to be a characteristic of long-lived families [19]. ACTH-induced Dehydroepiandrosterone (DHEA) has anti-aging properties and is considered to be related to longevity [20].

In this study, both the levels of ACTH and COR and their abnormal rates in patients with type 2 DM or BD were lower than those in patients with pre-diabetes. The incidence of the abnormal HPA axis decreased gradually from pre-diabetes to pre-diabetes comorbidity BD and then to the stage of type 2 DM or BD. This is consistent with the three-stages of ‘General Adaptation Syndrome’ proposed by previous scholars [21]. As mentioned above, the HPA axis is important for stress response, and glucocorticoids are involved in many functions of the body, such as immunity, energy metabolism, emotional regulation, and cognition [22]. Various stress factors can activate the HPA axis, leading to its hyperfunction, producing high levels of ACTH and glucocorticoids to help the body cope with stress. However, high levels of glucocorticoids could have adverse effects on the body, such as impaired cognitive function and emotional disorders [23]. The function of the HPA axis decreases with the continuous existence of stress, and it is difficult to produce enough cortisol to meet the needs of the body under stress. Therefore, in the stage of chronic stress, the levels of both ACTH and COR would decrease compared with the initial levels [24]. Besides, the levels of HPA axis hormones in patients among the three stages were all higher than those in controls, suggesting that the body activates the internal stress mechanism after the start of the rhythm disorder and supports the viewpoint of the ‘stress-dysrhythmia’ theoretical hypothesis that stress is involved in the occurrence of rhythm disorders.

The incidence of the abnormal HPT axis showed a trend of increasing moderately at first and then decreasing obviously from the stage of pre-diabetes to the stage of pre-diabetes comorbidity BD and then to the stage of type 2 DM or BD. It was thought that in chronic unpredictable stress response, hyperactive HPA axis function might inhibit the secretion of HPT axis hormones [25]. The high level of cortisol leads to dysfunction of the HPT axis, inhibiting the release of TSH in the pituitary and activities of thyroid peroxidase and oxidase, which then leads to an imbalance of negative feedback regulation among hormones in the HPT axis [26]. With the continuous existence of stress, the daily rhythms of TT3, TT4, and TSH showed a decreasing trend [27]. In this study, the increased abnormal rate of the HPT axis in the stage of pre-diabetes comorbidity BD suggested that with the failure of HPA axis function, the inhibition of the HPT axis was weakened, which was in accordance with the changes in neuroendocrine axis rhythms in the state of sustained stress. It also suggested, to some extent, that the process from pre-diabetes to type 2 DM was the process from local abnormal glucose metabolism rhythm to dysrhythmia. In addition, compared with controls, the levels of FT3 and FT4 were decreased in patients with abnormal glucose metabolism, and the level of TSH was increased in the initial stage of local abnormal rhythm, suggesting a gradual decreasing trend of HPT axis function with the progression of impaired glucose metabolism rhythm. Therefore, the detection of HPT axis hormone levels might contribute to the prediction of the severity of impaired body rhythms.

It is worth noting that BD (a typical emotional rhythm disorder) and type 2 DM were grouped together through cluster analysis, having similar changes in the neuroendocrine axes, which also verified that type 2 DM is a rhythm disorder. Meanwhile, the incidences of the abnormal neuroendocrine axes were all significantly higher in patients with both BD and abnormal glucose metabolism than in those with a single disease. This result suggested that impairments in the neuroendocrine axes were more severe when the body had multiple dysrhythmic activities.

For clinical safety aspects of reasons, this study did not include subjects with serious complications of DM, which may belong to the ‘loss of rhythm’ stage. In the future, these patients will be analysed to explore changes in neuroendocrine and other rhythmic staging markers in the final stage of dysrhythmia. Second, the sample size of the comorbidity group was small. This was based on real-world clinical observation data, and the comorbidity of abnormal glucose metabolism in patients with BD was consistent with the findings of a previous epidemiological study [28], which supports the reliability of our results to some extent. Third, considering the detection of a variety of neuroendocrine hormones, peripheral blood samples were collected uniformly. In our next study, salivary sequential cortisol or 24-hour urinary free cortisol data, which are more sensitive to HPA axis assessment, will be collected for analysis. Furthermore, since other metabolic disorders, such as dyslipidemia, may also occure as a result of the disturbance in circadian rhythm [29], we will include these indicators in future studies.

The overall prediction rate of the final regression model in this study reached 92.7%, and the prediction rate for the period of dysrhythmia (disease stage) reached 97.1%, which indicates that the 11 indexes included in the final regression model could accurately reflect the process of the continuous development of abnormal glucose metabolism rhythm of the body and had predictive value for evaluating the developmental stage of impaired glucose metabolism rhythm. These indicators may also be used as rhythm markers for the discussion of rhythm staging of other chronic non-infectious diseases in the future. It should be pointed out that this study aimed to serve as a preliminary observation. By exploring different profiles in different stages of blood glucose metabolic abnormality, we did not aim to define specific causal links. We only aimed to provide certain clinical clues for the rhythm staging of abnormal blood glucose metabolism; however, whether there is a causal relationship between them remains to be further explored.

In conclusion, this study provides some interesting and innovative basis to understand the pathological development of glucose metabolism rhythm more comprehensively from a holistic health perspective. It is an exploration of the application of psychosomatic medicine in the interdisciplinary field. It can be applied to the whole course management and early intervention of DM, as well as future development of optimised treatment based on rhythm regulation; thus, it has valuable clinical application.

## Supplementary Information

**Additional file 1: Supplementary table 1.** Comparisons of demographic data among the five groups. **Supplementary table 2.** Comparisons of the scores of five factors of NEO-FFI among the five groups. **Supplementary table 3.** Comparisons of hormonelevels and abnormal rates among the five groups. **Supplementary figure 1.** Comparisons of abnormal rates of neuroendocrine axes among the five case groups.

## Data Availability

All data generated or analysed during this study are included in this published article [and its supplementary information files].
