# Supplementary material for: Re-interpretation of the mechanism of type 2 diabetes mellitus based on a framework of psychosomatic medicine: a real-world study
Source: BMC Psychiatry. 2022 Nov 8;22:689. doi: 10.1186/s12888-022-04315-1 (PMC9644606; doi:10.1186/s12888-022-04315-1)
Supplement: Supplementary file 1 — Additional file 1: Supplementary table 1. Comparisons of demographic data among the five groups. Supplementary table 2. Comparisons of the scores of five factors of NEO-FFI among the five groups. Supplementary table 3. Comparisons of hormonelevels and abnormal rates among the five groups. Supplementary figure 1. Comparisons of abnormal rates of neuroendocrine axes among the five case groups. [file 12888_2022_4315_MOESM1_ESM.docx]

**Supplementary table 1: Comparisons of demographic data among the five groups**

| **groups**  M ± SD / N (%)  **variables** | | **BD group**  **(n = 134)** | **DM group**  **(n = 162)** | **Pre-DM group**  **(n = 77)** | **BD + DM group**  **(n = 14)** | **BD + pre-DM group**  **(n = 54)** | **F/χ^2^** | **P** |
| --- | --- | --- | --- | --- | --- | --- | --- | --- |
| Onset age (years) | | 24.37±12.506 | 53.67±11.493 | 37.95±11.391 | 30.43±6.501 | 27.57±10.930 | 116.586 | 0.000 |
| Age at enrolment (years) | | 30.54±14.230 | 59.92±10.622 | 42.86±11.482 | 39.71±11.248 | 34.56±13.130 | 132.164 | 0.000 |
| Confirmed time (months) | | 57.15 | 14.08 | 32.72 | 38.79 | 16.61 | 101.164 | 0.000 |
| sex | male | 24(17.9%) | 88(54.3%) | 37(48.1%) | 9(64.3%) | 22(40.7%) | 46.196 | 0.000 |
|  | female | 110(82.1%) | 74(45.7%) | 40(51.9%) | 5(35.7%) | 32(59.3%) |  |  |
| Address | town | 109(81.3%) | 122(75.3%) | 61(79.2%) | 9(64.3%) | 37(68.5%) | 5.286 | 0.259 |
|  | village | 25(18.7%) | 40(24.7%) | 16(20.8%) | 5(35.7%) | 17(31.5%) |  |  |
| Marriage | single | 86(83.5%) | 27(16.7%) | 14(18.2%) | 7(50%) | 27(50%) | 136.820 | 0.000 |
|  | married | 17(16.5%) | 135(83.3%) | 63(81.8%) | 7(50%) | 27(50%) |  |  |
| Educational level | ≤high school | 73(54.5%) | 126(77.8%) | 51(66.3%) | 5(36%) | 27(50%) | 28.224 | 0.000 |
|  | ≥junior college | 61(45.5%) | 36(22.2%) | 26(33.8%) | 9(64%) | 27(50%) |  |  |
| Stable income | no | 78(56.9%) | 32(19.8%) | 27(40.3%) | 2(14.3%) | 28(51.9%) | 51.343 | 0.000 |
|  | yes | 59(43.1%) | 130(80.2%) | 40(59.7%) | 12(85.7%) | 26(48.1%) |  |  |
| Comorbid hypertension | | 2(1.5%) | 84(51.9%) | 10(13.0%) | 4(28.6%) | 1(1.9%) | 129.771 | 0.000 |
| Family history | | 33(24.6%) | 44(27.2%) | 5(6.5%) | 7(50%) | 10(18.5%) | 20.274 | 0.000 |

**Supplementary table 2: Comparisons of** **the scores of five factors of NEO-FFI**

**among the five groups**

**factors**

| **v因**  **groups** | **Neuroticism** | **Extroversion** | **Openness** | **Agreeableness** | **Conscientiousness** |
| --- | --- | --- | --- | --- | --- |
| **BD group** | 34.79±8.173 | 35.70±6.908 | 37.15±3.616 | 46.28±4.141 | 42.64±5.885 |
| **DM group** | 24.65±5.211 | 39.32±6.482 | 34.49±3.292 | 48.8±3.351 | 48.8±5.743 ↑ |
| **Pre-DM group** | 36.9±7.434 | 34.46±6.754 | 32.69±5.581 | 36.31±7.884 | 33.26±10.026 ↓ |
| **BD + DM group** | 32.09±8.162 | 36.07±9.660 | 35.56±3.568 | 45.65±3.864 | 44.71±6.079 ↑ |
| **BD + pre-DM group** | 37.74±9.580 | 38.18±9.147 | 37.11±5.958 | 40.17±4.575 | 37.28±6.251 |
| **The Norm** | 20.4-38.8 | 26-42 | 32-47 | 30-48 | 36-44 |
| **F** | 62.913 | 8.218 | 12.471 | 103.434 | 78.827 |
| **P** | 0.000 | 0.000 | 0.000 | 0.000 | 0.000 |

**Supplementary table 3: Comparisons of hormone levels and abnormal**

**rates among the five groups**

| **groups**  **variables** | | **BD group** | **DM group** | **Pre-DM group** | **BD + DM** | **BD + pre-DM** | **F/χ^2^** | **P** |
| --- | --- | --- | --- | --- | --- | --- | --- | --- |
| TSH | levels | 2.20±1.301 | 2.14±2.012 | 2.48±1.537 | 2.04±1.271 | 2.61±3.399 | 11.745 | 0.019 |
|  | AR* | 6.00% | 7.40% | 9.10% | 7.10% | 14.80% | 4.278 | 0.370 |
| TT3 | levels | 1.43±0.299 | 1.43±0.510 | 1.64±0.575 | 1.33±0.252 | 1.50±0.477 | 16.991 | 0.002 |
|  | AR | 3.70% | 11.70% | 7.80% | 7.10% | 37.00% | 43.642 | 0.000 |
| FT3 | levels | 4.37±0.680 | 4.07±1.422 | 4.70± 1.214 | 4.05±0.611 | 4.49±1.108 | 32.101 | 0.000 |
|  | AR | 3.00% | 13.00% | 19.50% | 0% | 16.70% | 18.420 | 0.001 |
| TT4 | levels | 88.42±21.080 | 91.39±24.615 | 105.51±32.832 | 79.29±23.728 | 67.59±37.517 | 40.271 | 0.000 |
|  | AR | 4.50% | 6.20% | 6.50% | 7.10% | 27.80% | 30.502 | 0.000 |
| FT4 | levels | 12.34±1.691 | 13.08±3.748 | 14.70±4.129 | 12.52±1.770 | 13.36±9.572 | 20.006 | 0.000 |
|  | AR | 1.50% | 5.60% | 18.20% | 7.10% | 31.50% | 48.931 | 0.000 |
| AR of HPT axis | | 15.70% | 26.50% | 39% | 21.40% | 57.40% | 37.281 | 0.000 |
| ACTH | levels | 25.71±12.950 | 33.88±14.455 | 36.79±33.077 | 35.59±20.175 | 42.52±33.421 | 29.199 | 0.000 |
|  | AR | 7.50% | 11.10% | 18.20% | 28.60% | 7.40% | 10.564 | 0.032 |
| COR | levels | 368.78±162.468 | 442.13±168.596 | 656.74±311.641 | 378.82±195.414 | 577.35±234.725 | 87.083 | 0.000 |
|  | AR | 9.00% | 6.80% | 39.00% | 21.40% | 35.20% | 57.266 | 0.000 |
| AR of HPA axis | | 16.40% | 16.70% | 45.50% | 50.00% | 35.20% | 36.154 | 0.000 |
| AR of biax | | 0.70% | 4.90% | 14.30% | 14.30% | 22.20% | 32.395 | 0.000 |

*: abnormal rates

**Supplementary figure 1:** **Comparisons of abnormal rates of neuroendocrine axes**

**among the five case groups**
